# HIV-1 protease-induced apoptosis

**DOI:** 10.1186/1742-4690-11-37

**Published:** 2014-05-20

**Authors:** Michaela Rumlová, Ivana Křížová, Alena Keprová, Romana Hadravová, Michal Doležal, Karolína Strohalmová, Iva Pichová, Miroslav Hájek, Tomáš Ruml

**Affiliations:** 1 Institute of Organic Chemistry and Biochemistry, Academy of Sciences of the Czech Republic, v.v.i., IOCB & Gilead Research Center, Flemingovo nám. 2, 166 10 Prague, Czech Republic; 2 Department of Biochemistry and Microbiology, Institute of Chemical Technology Prague, Technická 5, 166 28 Prague, Czech Republic; 3 Department of Biotechnology, Institute of Chemical Technology Prague, Technická 5, 166 28 Prague, Czech Republic

**Keywords:** HIV protease, BCA3, AKIP-1, Apoptosis, Mitochondria

## Abstract

**Background:**

Apoptosis is one of the presumptive causes of CD4^+^ T cell depletion during HIV infection and progression to AIDS. However, the precise role of HIV-1 in this process remains unexplained. HIV-1 protease (PR) has been suggested as a possible factor, but a direct link between HIV-1 PR enzymatic activity and apoptosis has not been established.

**Results:**

Here, we show that expression of active HIV-1 PR induces death in HeLa and HEK-293 cells *via* the mitochondrial apoptotic pathway. This conclusion is based on *in vivo* observations of the direct localization of HIV-1 PR in mitochondria, a key player in triggering apoptosis. Moreover, we observed an HIV-1 PR concentration-dependent decrease in mitochondrial membrane potential and the role of HIV-1 PR in activation of caspase 9, PARP cleavage and DNA fragmentation. In addition, *in vitro* data demonstrated that HIV-1 PR mediates cleavage of mitochondrial proteins Tom22, VDAC and ANT, leading to release of AIF and Hsp60 proteins. By using yeast two-hybrid screening, we also identified a new HIV-1 PR interaction partner, breast carcinoma-associated protein 3 (BCA3). We found that BCA3 accelerates p53 transcriptional activity on the *bax* promoter, thus elevating the cellular level of pro-apoptotic Bax protein.

**Conclusion:**

In summary, our results describe the involvement of HIV-1 PR in apoptosis, which is caused either by a direct effect of HIV-1 PR on mitochondrial membrane integrity or by its interaction with cellular protein BCA3.

## Background

A major determinant of pathogenicity in untreated human HIV-1 infection is depletion of CD4^+^ T cells, caused in large part by apoptosis (reviewed in [1-4]). Six HIV-1-encoded proteins—surface glycoprotein (gp120), Tat, Nef, Vpr, Vpu and protease (PR)—have been shown to induce apoptosis [1-3,5-8]. During HIV-1 infection, the virus-encoded aspartic PR is expressed as part of the Gag-Pol polyprotein precursor. The Gag polyprotein comprises the structural proteins of the viral shell (matrix, capsid and nucleocapsid), while the Pol polyprotein consists of viral PR, reverse transcriptase and integrase. Upon expression, the polyprotein precursors Gag and Gag-Pol migrate to the plasma membrane, where they assemble into immature viral particles. During or shortly after budding of an immature HIV-1 particle, the protease self-activates and is released from its precursor. The active PR then cleaves polyprotein precursors into mature proteins. Despite widely accepted opinion that retroviral proteases are activated during virus budding, specific processing of HIV-1 polyproteins has been identified also in the membrane fraction and cytoplasm of HIV-1-infected cells, suggesting that some PR activation takes place within the cells before Gag reaches the plasma membrane [9].

Expression of HIV-1 PR is cytotoxic for transfected cells [10-12]. Although several cellular proteins, such as actin [13], laminin [14], vimentin [15,16], Bcl-2 [11], eukaryotic initiation factor 4G [17], procaspase 8 [18-20], PABPC1 [21] and a subunit of eukaryotic translation factor 3 (eIF3d) [22], can be cleaved by HIV-1 protease, the precise mechanism of cell death caused by HIV-1 PR is still unclear. The first suggested mechanism was direct cleavage of the anti-apoptotic protein Bcl-2 by HIV-1 PR, leading to cell death *via* apoptosis [11]. However, this was not confirmed in subsequent studies [12,18,22]. Morphological changes typical of necrosis—but no apoptosis activation—have been observed in COS7 cells expressing HIV-1 PR [12]. Another mechanism proposed for HIV-1 PR-mediated apoptosis was based on the observation that PR cleaves caspase 8 both *in vitro* and *in vivo* to yield a novel Casp8p41 fragment [18,19]. Two mechanisms of potential Casp8p41-initiated caspase-dependent cell death have been proposed: (i) Casp8p41 mediates activation of pro-apoptotic protein Bid by cleaving it to form tBid [18] and (ii) Casp8p41 independently induces apoptosis by interacting with mitochondria, resulting in rapid Bax/Bak mediated mitochondrial depolarization [19,23,24]. In both proposed mechanisms, cytochrome c is released from mitochondria, caspase 9 and 3 are activated and the cell is directed to its death.

Breast carcinoma-associated protein 3 (BCA3, a proline-rich protein also known as A-kinase interacting protein or AKIP1) was first identified and characterized in breast and prostate cancer cell lines [25,26]. The full-length transcript consists of six exons, with a translation start in exon 2. However, several BCA3 splice variants have been identified in various cell lines [26,27]. Although BCA3 has been reported to interact with various cellular proteins (e.g. cAMP-dependent protein kinase A (PKAc) [27], NFκB [28-30], p73 [31], apoptosis inducing factor (AIF) [32]) its precise cellular role remains unclear.

Here, we report that BCA3 acts as a novel HIV-1 PR binding partner. We show that HIV-1 PR is localized in the mitochondria of HEK-293 and HeLa cells, where it interacts with BCA3. We also show that expression of HIV-1 PR in HEK-293 and HeLa cells most likely leads to cell death *via* the intrinsic mitochondrial apoptotic pathway. We based this conclusion on several observations: (i) direct localization of HIV-1 PR in the mitochondria; (ii) *in vitro* cleavage of mitochondrial proteins, such as the receptor for the outer membrane translocase complex (Tom22), voltage-dependent anion channel (VDAC) and adenine nucleotide translocator (ANT), leading to release of pro-apoptotic mitochondrial proteins; (iii) HIV-1 PR concentration-dependent decrease in mitochondrial membrane potential (ΔΨ_m_) and (iv) activation of caspase 9 (Casp-9), poly-(ADP-ribose)-polymerase (PARP) cleavage and DNA fragmentation. HIV-1 PR-induced apoptosis is enhanced by BCA3, which enhances p53 transcriptional activity on the *bax* promoter.

## Results

### HIV-1 PR interacts with BCA3

**Figure 1:**
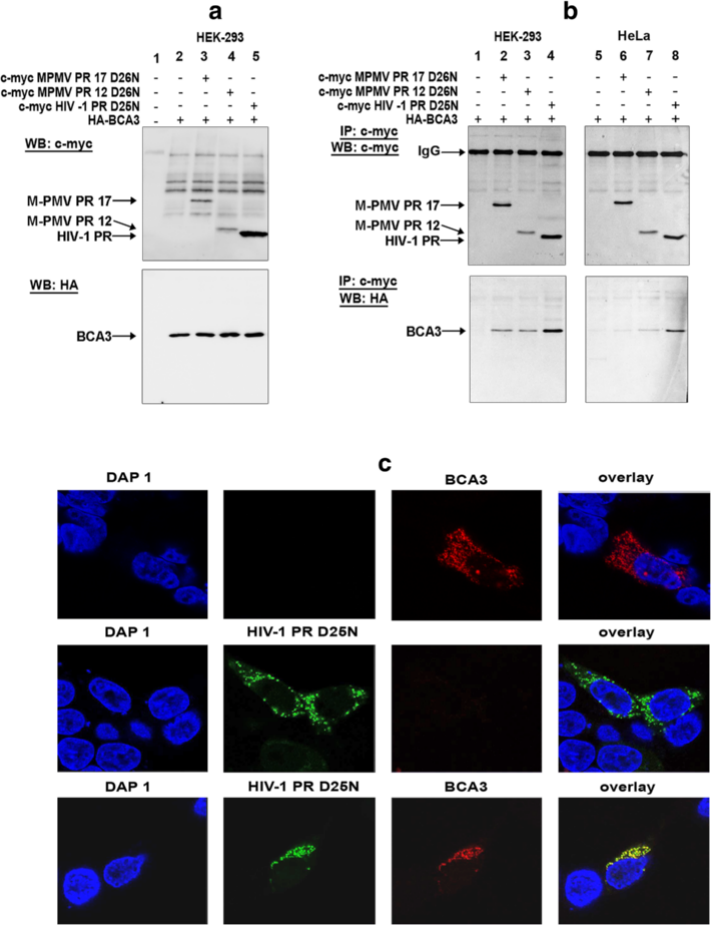
**Interaction of M-PMV PR, HIV-1 PR and BCA3. (a)** Western blot analysis of total lysates of the HEK-293 cells transfected with *c-myc-M-PMV PR17(D26N)*, *c-myc-M-PMV PR12(D26N)*, *c-myc-HIV-1 PR(D25N)* and *HA-BCA3*. The blots were developed with anti c-myc (upper panel) and HA (lower panel) antibodies. **(b)** Co-immunoprecipitation of M-PMV or HIV PRs with BCA3: HEK-293 (lanes 1–4) and HeLa (lanes 5–8) cells transfected with *c-myc-M-PMV PR17(D26N)*, *c-myc-M-PMVPR12(D26N)* or *c-myc-HIV-1 PR(D25N)* together with *HA-BCA3* were lysed 48 h post-transfection, and proteins were immunoprecipitated using anti-c-myc antibody. Precipitates were blotted and developed with anti-c-myc (upper panel) or anti-HA (lower panel) antibodies. **(c)** Immunocolocalization of HIV-1 PR(D25N) with BCA3 in HEK-293 cells using confocal microscopy: HEK-293 cells transfected with *c-myc-HIV-1 PR(D25N)* together with *HA-BCA3* were stained 48 h post-transfection using anti HA-TRITC or c-myc-FITC antibodies and visualized using confocal microscopy. The two black panels represent the samples of the cells not producing BCA3 (first row) and HIV-1 PR (second row) demonstrating thus that HA-TRITC and c-myc-FITC antibodies do not provide non-specific signal.

Using a yeast two-hybrid system, we identified BCA3 as a novel binding partner of Mason-Pfizer monkey virus protease (M-PMV PR) [33]. As three-dimensional structures of retroviral proteases share certain conserved features [34,35], we analyzed the ability of catalytically inactive HIV-1 PR(D25N) to bind to BCA3. Co-immunoprecipitation experiments of the cell expressing M-PMV or HIV-1 PRs together with BCA3 (Figure 1a) clearly showed that besides both forms of M-PMV PRs, i.e., PR17(D26N) and PR12(D26N), (Figure 1b, lanes 2,3,6,7) also HIV-1 PR(D25N) directly interacts with BCA3 in HeLa and HEK-293 cells (Figure 1b, lanes 4,8).

We used confocal microscopy to confirm these results (Figure 1c). Immunofluorescence staining showed that HA-BCA3 was localized mainly in the cytoplasm and partially within the nucleus of HEK-293 cells, whereas HIV PR was localized only within the cytosol in a speckled pattern. Co-expressed BCA3 and HIV-1 PR co-localized in the cytoplasm (Figure 1c, lower panels).

### Mitochondrial localization of BCA3 and HIV PR

**Figure 2:**
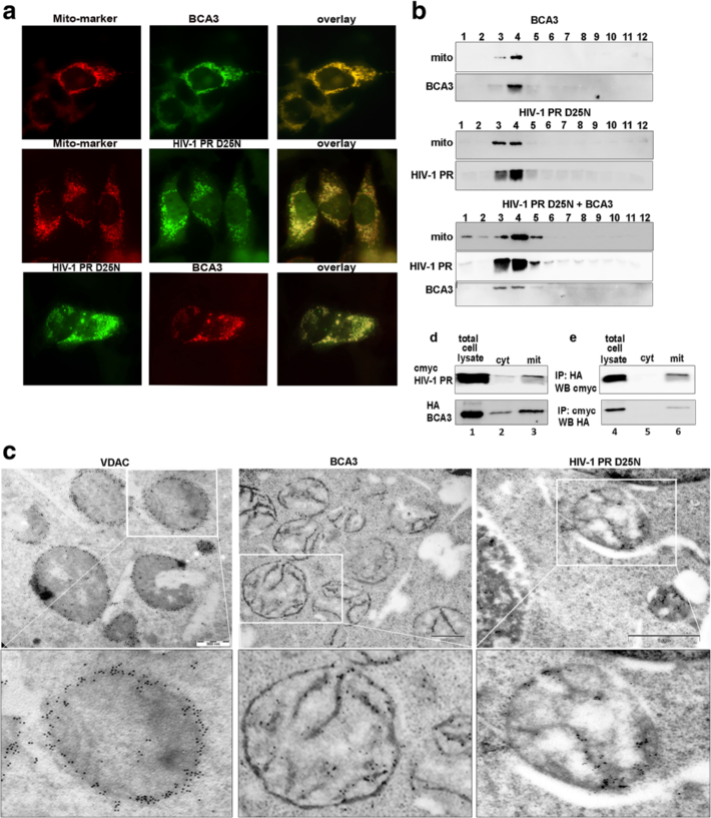
**Mitochondrial localization of HIV-1 PR and, BCA3. (a)** HeLa cells were co-transfected with *c-myc-HIV-1 PR(D25N)*, or *HA-BCA3* and mitochondrial marker *pDsRed2-Mito*. HeLa cells were stained 48 h post-transfection using anti-HA-TRITC or c-myc-FITC antibodies and visualized using confocal microscopy. Mitochondria were stained red; the overlaid images show colocalization of mitochondrial marker with BCA3 and HIV-1 PR(D25N), as well as HIV-1 PR(D25N) with BCA3. **(b)** Purified mitochondria from HeLa cells transfected with *c-myc-HIV-1 PR(D25N)*, *HA-BCA3*, or a combination of *c-myc-HIV-1 PR(D25N) with HA-BCA3*. Fractions from Optiprep gradient (labeled 1–12) were analyzed using Western blot with Mitochondrial Marker antibody and anti-HA and anti-c-myc antibodies. **(c)** Immunogold TEM analysis of localization of VDAC (left panels, bar represents 500 nm), BCA3 (middle panels, bar represents 500 nm) and HIV-1 PR(D25N) (right panels, bar represents 1 μm) in HEK-293 cells. **(d,e)** Western blot **(d)** and co-immunoprecipitation followed by Western blot analysis **(e)** of HIV-1 PR(D25N) and BCA3 present in cytosolic and mitochondrial fractions. HEK-293 cells (4x10^5^ cells/ml) were co-transfected with *HA-BCA3* and *c-myc-HIV-1 PR(D25N)*. After 48 h, the cells were washed 1× with PBS and 1/20 of the cells was resuspended in PLB and used as a total cell lysate sample for Western blot analysis (**d**, lane 1). 1/4 of the cells were used for immunoprecipitation (**e**, lane 4) using anti-HA and anti-c-myc antibodies. The last 3/4 of the cells was used for the isolation of cytosolic and mitochondrial fractions. 100 μl aliquots of both cytosolic and mitochondrial fractions were analyzed using Western blot (**d**, lanes 2 and 3, respectively) and 900 μl aliquots of both cytosolic and mitochondrial fractions samples were used for immunoprecipitation using anti-HA and anti-c-myc antibodies and developed by Western blot (**e**, lanes 5 and 6).

We next analyzed whether localization of HIV-1 PR – BCA3 complex corresponds to a particular subcellular compartment. HeLa cells were co-transfected with expression vectors encoding mitochondrial (*Mito-GFP*, *Mito-dsRed*) or endoplasmic reticulum (*ER-GFP*) markers and *HA-BCA3* or *c-myc-HIV-1 PR(D25N)*. Localization of the proteins was analyzed using fluorescent microscopy. Both BCA3 and HIV-1 PR(D25N) proteins were identified in the mitochondria (Figure 2a). To further confirm their mitochondrial localization, HEK-293 or HeLa cells co-expressing *HA-BCA3* and *c-myc-HIV-1 PR(D25N)* were analyzed. Western blot analysis confirmed the presence of BCA3 and HIV-1 PR(D25N) in the main mitochondrial fraction of iodixanol gradient (Figure 2b). Immunogold transmission electron microscopy (TEM) also confirmed the presence of both BCA3 and HIV-1 PR(D25N) mainly at the mitochondria (Figure 2c). In contrast to the control mitochondrial protein voltage-dependent anion channel (VDAC), which was present exclusively at the mitochondrial surface, the BCA3 and HIV-1 PR(D25N) proteins were also localized at membrane invaginations reminiscent of cristae. Co-immunoprecipitation of BCA3 with HIV-1 PR(D25N) from mitochondrial fractions confirmed the interaction of these two proteins in the mitochondrial, but not in the cytosolic fractions (Figure 2d,e).

Detailed analysis of the composition of the mitochondrial fractions containing BCA3 and HIV-1 PR(D25N) verified the presence of several mitochondrial proteins, including Mito-marker (Abcam), VDAC, cytochrome C (COXIV), receptor of the outer membrane translocase complex (Tom22) and Bax (Additional file 1: Figure S1a). We also verified that the mitochondrial localization of HIV-1 PR(D25N) is not an artifact caused by structural changes introduced either by the active site mutation (D25N) or the N-terminal extension by c-myc/HA tags. The association of these HIV-1 protease variants with mitochondria was confirmed by Western blot analysis of mitochondria isolated from cells producing inactive (D25N) and active HIV-1 PR, and N-terminally extended PR with native flanking sequence, i.e. HIV-1 PRO in the presence of ritonavir (Additional file 1: Figure S1b,c). The presence of HIV-1 PR(D25N)/BCA3 complex in endoplasmic reticulum was excluded (Additional file 1: Figure S1d). Next we verified that the HIV-1 PR interacts also with endogenous BCA3. Because the endogenous level of expression of BCA3 is under detection limit of commercially available antibodies, we prepared stably transfected 293-HEK cell line (labelled D1) in which the level of HA-BCA3 expression is significantly lower than in transiently transfected cells. These cells were transfected with *c-myc-HIV-1 PR (D25N)* or *c-myc-HIV-1 PRO (D25N)* and co-immunoprecipitations using c-myc and HA antibodies were carried out. Western blot analysis confirmed that both HIV-1 PR and HIV-1 PRO interacted with BCA3 present at low intracellular level (Additional file 1: Figure S1e).

### Sublocalization of HIV-1 PR(D25N)/BCA3 complex in mitochondria

**Figure 3:**
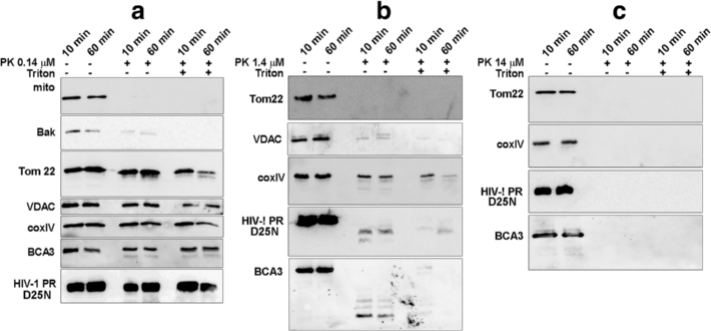
**OMM localization of HIV-1 PR and BCA3.** Purified mitochondria from HEK-293 cells co-transfected with *c-myc-HIV-1 PR(D25N)* and *HA-BCA3* were incubated with 0.14 μM **(a)**, 1.4 μM **(b)** or 14 μM **(c)** proteinase K in the presence or absence of Triton X-100 for 10 and 60 min on ice. The reaction was stopped by the addition of 6× protein loading buffer (PLB), and samples were analyzed by Western blotting.

To define the submitochondrial localization of the HIV-1 PR/BCA3 complex, mitochondria isolated from HEK-293/HeLa cells transfected with *c-myc-HIV-1PR(D25N)* and *HA-BCA3* were treated with increasing amounts of proteinase K (PK; 0.14, 1.4, and 14 μM) in the presence or absence of Triton X-100. We also determined the accessibility of PK to selected proteins localized: i) at the cytoplasmic site of the outer mitochondrial membrane (OMM), i.e., Bak, Mito-marker (Abcam) and Tom22; ii) in the OMM, i.e., VDAC; and iii) in the inner mitochondrial membrane (IMM), i.e., COXIV. At the lowest concentration, PK completely degraded Bak and Mito-marker (Abcam), whereas Tom22, which is likely to be protected by its close association with other Tom proteins was partially degraded only in the presence of detergent (Figure 3a). However, at the higher concentration (1.4 μM), PK efficiently degraded not only Tom22 but also VDAC (Figure 3b). The IMM-localized COXIV protein was resistant, and its complete degradation by PK was observed only at the highest PK concentration (Figure 3c). Similarly, BCA3 and HIV-1 PR(D25N) were degraded only at PK concentrations of 1.4 μM and higher (Figure 3b), suggesting their localization inside in the mitochondria. This result is in agreement with the immunogold TEM (Figure 2c) which also suggested that HIV-1 PR(D25N) and BCA3 are associated with the inner mitochondrial membrane.

### Expression of HIV-1 PR triggers mitochondrial apoptosis

**Figure 4:**
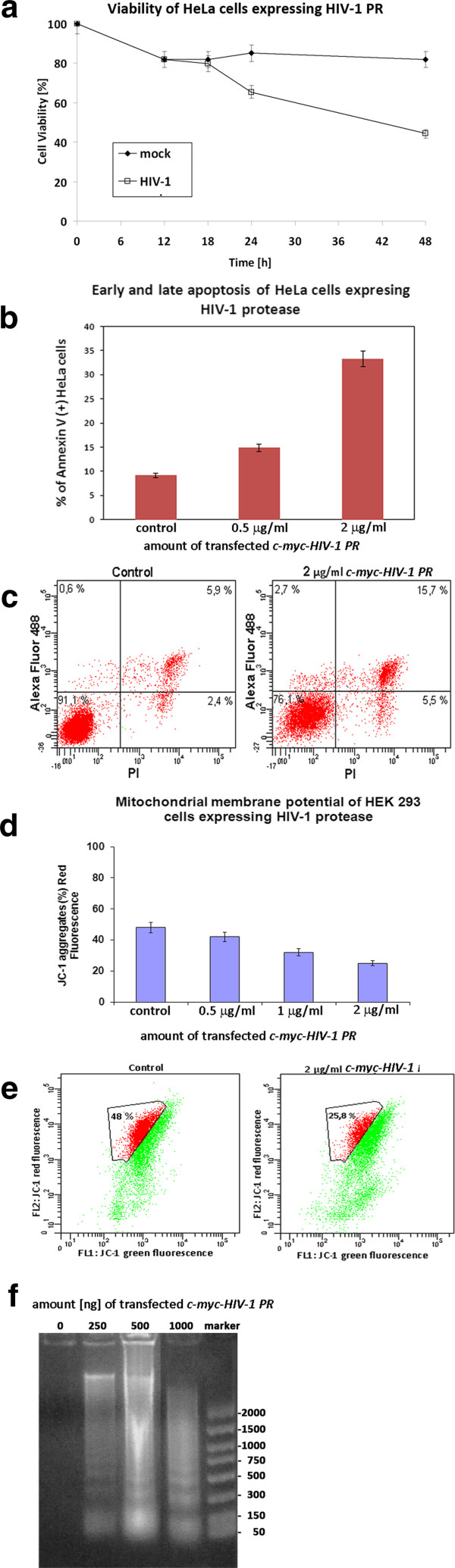
**Apoptotic effect of HIV-1 PR. (a)** Cytotoxic effect of HIV-1 PR on HeLa cells. HeLa cells were transfected with *c-myc-HIV-1 PR* (1 μg/ml), encoding catalytically active HIV-1 PR and the number of dead cells at various post-transfection time points was assessed by PI staining by flow cytometry using BD LSR Fortessa. **(b)** HeLa cells were transfected with indicated amounts of *c-myc-HIV-1 PR*. Six hours post-transfection, the cells were labeled with FITC Alexa Fluor® 488 annexin V and propidium iodide (PI), and the apoptotic + dead cells were detected using BD LSR Fortessa. **(c)** Representative gating for control and HIV-1 PR expressing cells. **(d)** The HEK-293 cells were transfected with varying amounts of *c-myc-HIV-1 PR*. Changes in ΔΨ_m_ were determined six hours post-transfection by staining with JC-1 fluorochrome followed by flow cytometry using BD LSR Fortessa. **(e)** Representative gating for control and HIV-1 PR expressing cells. Healthy cells containing red JC-1 aggregates were detected in the FL 2 channel, whereas green JC-1 monomers in apoptotic cells were monitored in the FL1 channel (FITC). **(f)** HEK-293 cells were transfected with *c-myc-HIV-1 PR*, and 24 h post-transfection, fragmented DNA was isolated using the Suicide-Track^TM^ DNA Ladder Isolation Kit and separated by agarose gel. The mean percentage (±standard deviation) of the data pooled from three independent experiments is shown. Each data point was repeated at least twice.

To determine whether the increased cell death induced by expression of catalytically active HIV-1 PR (Figure 4a) can be attributed to apoptosis, either HeLa or HEK-293 cells were transfected with various amounts of *c-myc-HIV-1 PR*, and their progression to apoptosis was determined as described in Materials and Methods. Six hours post-transfection, we observed an HIV-1 PR concentration-dependent increase of a number of annexin-V-positive apoptotic cells (Figure 4b,c) and a protease-concentration-dependent decrease in mitochondrial membrane potential ΔΨ_m_ (Figure 4d,e). We also observed fragmentation of nuclear DNA in HEK-293 cells transfected with *c-myc-HIV-1 PR*, characteristic of the late phase of apoptosis (Figure 4f).

**Figure 5:**
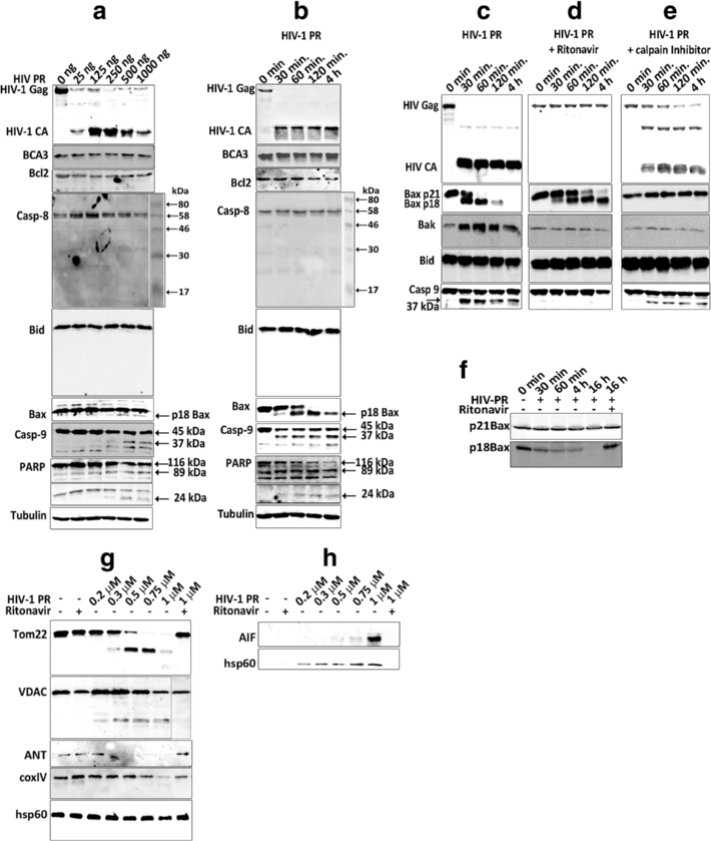
**Proteolytic processing of cellular proteins by active HIV PR. (a)** HEK-293 cells transfected with *HIV-1 Gag* and *HA-BCA3* (500 ng each) and various amounts of *c-myc-HIV-1 PR* (25–1000 ng) were analyzed 48 h post-transfection. **(b-e)** HEK-293 cells transfected with *HIV-1 Gag* and *HA-BCA3* were lysed 48 h post-transfection as described in Materials and Methods and incubated with recombinant HIV-1 PR (1.25 μM), optionally in the presence of ritonavir (4 μM) **(d)** or calpain inhibitor I (100 μM) **(e)** at 37°C for the time indicated. The proteins were then separated on 12-15% SDS-PAGE and analyzed by Western blot. **(f)** Autoradiographic analysis of HIV-1 PR *in vitro* cleavage of full-length p21Bax and N-terminally truncated p18Bax proteins obtained by TNT transcription/translation system. Protein samples were treated with HIV-1 PR (1.25 μM), optionally in the presence of ritonavir (8 μM), at room temperature for the time indicated. The proteins were then separated on 15% SDS-PAGE and visualized by autoradiography. **(g)***In vitro* cleavage of mitochondrial proteins by HIV-1 PR. Freshly isolated mitochondria from HEK-293 cells were incubated with various amounts of recombinant HIV-1 PR, optionally in the presence of ritonavir (4 μM). The proteins in the samples were separated on 12-15% SDS-PAGE and analyzed by Western blot. **(h)** Supernatant after cleavage of proteins from purified mitochondrial fraction. The samples from **(g)** were centrifuged, and supernatants were analyzed by Western blot.

Our finding that HIV-1 PR is associated with mitochondria and causes apoptosis accompanied with a loss of ΔΨ_m_ suggested its direct involvement in mitochondrial membrane permeabilization (MMP), typical for the intrinsic mitochondrial pathway of apoptosis. Therefore, we next analyzed the impact of HIV-1 PR on selected anti- and pro-apoptotic Bcl-2 family proteins involved in mitochondrial outer membrane permeabilization (MOMP) using: (i) *in vivo* cleavage of cellular proteins in HeLa or HEK-293 cells producing catalytically active HIV-1 PR (Figure 5a) and (ii) *in vitro* cleavage of whole HeLa or HEK-293 cell extracts by recombinant HIV-1 PR (Figure 5b). First we confirmed that PR was active, as its natural substrate Gag polyprotein was efficiently cleaved to capsid protein (CA) both in cells transfected with *c-myc-HIV-1 PR* (Figure 5a) and in cell lysates by using recombinant PR (Figure 5b). The profile of HIV-1 PR-mediated cleavage of selected cellular proteins was similar for both approaches (compare Figure 5a and b). The BCA3 protein was not degraded by HIV-1 protease either *in vitro* or *in vivo*. Neither had we observed any cleavage of anti-apoptotic molecule Bcl-2, pro-caspase 8 cleavage to Casp8p41 nor Bid truncation to tBid. In contrast, both *in vivo* and *in vitro*, we identified the activation of pro-caspase 9 to caspase 9 (Casp-9) (Figure 5a,b) which occurs only in response to cytochrome c release from mitochondria. Cleavage of poly(ADP-ribose) polymerase (PARP), occurring in the late phase of apoptosis upon activation of caspase 3, was also observed (Figure 5a,b). Moreover, we identified *in vitro* cleavage of the pro-apoptotic protein Bax (p21) to its lower molecular weight form (Figure 5b). It has been shown that such N-terminal cleavage of p21Bax, mediated by cysteine protease calpain to yield p18Bax, occurs during stress-induced apoptosis [36-39]. To study whether HIV-1 PR may also trigger or enhance Bax cleavage, either HEK-293 or HeLa cell extracts were treated with recombinant HIV-1 PR, with or without an HIV-1 protease-specific inhibitor (ritonavir) or calpain protease inhibitor I. In the cell lysate treated with recombinant HIV-1 PR, Bax was processed to its p18Bax form and then degraded during long incubations (Figure 5c, lane 4 h). Ritonavir that efficiently blocked HIV-1 PR (Figure 5d) did not inhibit calpain protease, (Figure 5d). However, ritonavir blocked complete degradation of p18Bax by residual calpain from the cell lysate (compare lanes corresponding to 4 h of incubation in Figure 5c,d). Calpain protease inhibitor I did not inhibit Gag processing by HIV-1 PR but completely blocked Bax cleavage (Figure 5e). No HIV-1 PR cleavage of Bak or Bid proteins was observed. Interestingly, activation of caspase 9 occurred only in samples containing active HIV-1 PR (Figure 5c,e). This data suggests that, under *in vitro* conditions, calpain protease cleaves p21Bax to p18Bax and HIV-1 PR subsequently cleaves p18Bax but not p21Bax protein. To verify this hypothesis, the *bax* gene was isolated from a HeLa cell cDNA library and genes encoding wild-type p21Bax and p18Bax were cloned under transcriptional control of *T7* bacteriophage promoter. Both proteins were synthesized using an *in vitro* transcription/translation system and subjected to proteolysis by HIV-1 PR in the presence or absence of ritonavir. In contrast to p21Bax, which was not cleaved by HIV-1 PR, the truncated p18Bax was degraded (Figure 5f).

The activation of caspase 9 and cleavage of PARP were the only changes directly affected by the presence of HIV-1 PR (Figure 5a-e). We next analyzed whether there is any direct proteolytic action of HIV-1 PR on purified mitochondria. Isolated mitochondria were treated with various amounts of recombinant HIV-1 PR, and selected mitochondrial proteins were analyzed by Western blot (Figure 5g). We observed increased proteolytic processing of the OMM proteins such as Tom22 and VDAC, as well as IMM-localized ANT in an HIV-1 PR concentration-dependent manner. To identify whether HIV-1 PR causes release of mitochondrial proteins involved in apoptosis, purified mitochondria were incubated with various amounts of HIV-1 PR in the presence or absence of ritonavir. The mitochondria were then pelleted and selected proteins released into supernatant were analyzed by Western blot. Specifically, apoptotic inducing factor (AIF) and Hsp-60 were released into the supernatant (Figure 5h).

### BCA3 enhances transcriptional activity of p53 on the bax promoter

**Figure 6:**
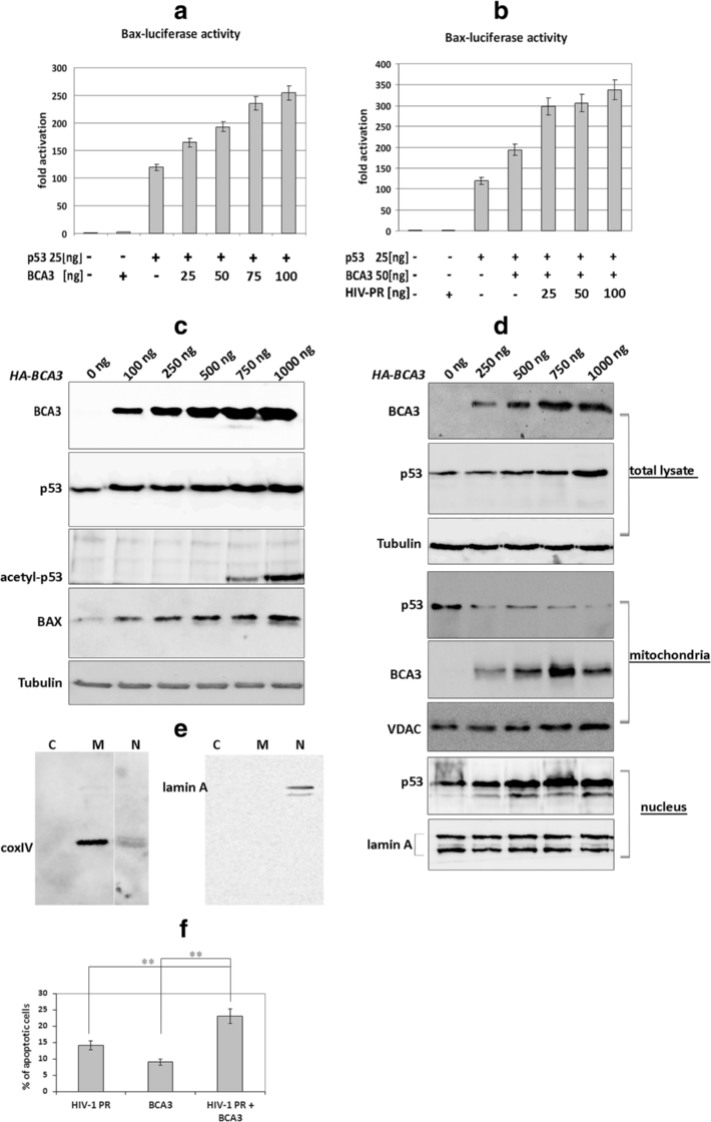
**BCA3 modulates p53 transcriptional activity.** Analysis of *bax*-promoter-Luciferase reporter activation in response to dose of *HA-BCA3***(a)**, *HA-BCA3* with *c-myc-HIV-1 PR(D25N)***(b)** transiently transfected in HEK-293 cells. **(c)** Endogenous levels of p53 and Bax in the extract of the HEK-293 cells transfected with increasing amounts of *HA-BCA3*. **(d)** Subcellular fractionation and detection of endogenous p53 from HEK-293 cells expressing increasing amounts of BCA3. **(e)** Protein control of cytosolic (C), mitochondrial (M) and nuclear (N) fractions purity: fractions were purified as described in Material and Methods and analyzed using antibody against cytochrome c (mitochondrial marker) and lamin A (nuclear marker) antibodies. **(f)** Mean percentage of apoptotic, Anexin-V positive cells expressing HIV-1 PR, BCA3 or both. Each experiment was performed in triplicate; the mean values of three independent experiments (with standard deviation) are shown. The p value was insignificant (p < 0.4700295) between HIV-1 PR and BCA3, represented by columns 1 and 2, respectively. HIV-1 PR + BCA3 expression on apoptosis was statistically significant, characterized by p < 0.0047547 and p < 0.0015748 for the cells expressing HIV-1 PR and BCA3, respectively.

BCA3 affects the activity of transcription factor p73, which is a p53 homologue [31]. As the p53 tumor suppressor is known to be a direct activator of the *bax* gene promoter [11,40], we examined whether BCA3 could regulate the *bax* promoter by modulating its transcription by p53. The *pGL3-bax-luc* reporter vector encoding a *luciferase* gene under control of the *bax* promoter was co-transfected into HEK-293 cells with 25 ng of *p53* plasmid DNA and various amounts of *HA-BCA3* plasmid (25–100 ng). Luciferase activity was measured 24–48 h post-transfection. As shown in Figure 6a, BCA3 had a dose-dependent activation effect on p53-induced transcriptional activity of *bax* promoter. Addition of *c-myc-HIV-PR(D25N)* increased *bax* promoter activation compared to expression of BCA3 alone (Figure 6b). To analyze whether BCA3 could activate endogenous p53 and thus enhance the level of Bax protein, we performed Western blot analysis of HEK-293 cells transfected with various amounts of *HA-BCA3*. BCA3 enhanced expression of p53, Bax and an acetylated form of p53 [41] (Figure 6c). As it is well-established that p53 rapidly translocates to mitochondria in response to stress signals [42], we isolated and analyzed nuclear and mitochondrial fractions from HEK-293 cells expressing increasing amounts of BCA3. As expected, the BCA3 protein was identified in isolated mitochondrial fractions (Figure 6d,e). However, we did not observe increased concentration of p53 in the mitochondrial fraction, but rather in the nuclear fraction (Figure 6d). Apoptosis was increased in the cells co-transfected with *c-myc-HIV-1 PR* and *HA-BCA3* in contrast to those transfected with only *c-myc-HIV-1 PR* or *HA-BCA3* (Figure 6f). The *p* value was calculated by using Tukey Honest Significant Difference test. The test has shown insignificant difference (p < 0.47) between HIV-1 PR and BCA3 (columns 1 and 2 in Figure 6f). Importantly, the effect HIV-1 PR + BCA3 expression on apoptosis was statistically significant, characterized by p < 0.0047547 and p < 0.0015748 for the cells expressing HIV-1 PR and BCA3, respectively. Thus, BCA3 seems to contribute to apoptosis induced by HIV-1 PR.

## Discussion and conclusions

It has been suggested that depletion of CD4^+^ T cells induced by HIV-1 is associated with a change in mitochondrial permeability that leads to cellular apoptosis [5,43-46].

Several HIV-1 proteins, such as surface glycoprotein (gp120), Tat, Nef, Vpr, Vpu and PR, induce apoptosis by triggering mitochondrial outer membrane permeabilization (MOMP), thereby causing cell death [1-3,47]. The cytotoxic effect of HIV-1 PR in bacterial, yeast and human cells has been attributed to its proteolytic activity [10-12]. However, precise mechanisms of either necrosis or apoptosis in HIV-1 PR-expressing cells have not been uncovered. There are two prominent molecular pathways of apoptosis: the extrinsic pathway, also called the “death receptor pathway;” and the intrinsic pathway, known as the “mitochondrial pathway.” Most cell deaths in vertebrates proceed *via* the intrinsic pathway, in which apoptosis is triggered by an intracellular cascade of events in which MOMP regulated by Bcl-2 family proteins plays a crucial role [4,48].

Here, we show that the expression of HIV-1 PR in HEK-293 or HeLa cells leads to the mitochondrial apoptotic pathway. It was previously reported that HIV-1 PR induces apoptosis *via* proteolytic cleavage of two proteins involved in regulation of apoptosis, Bcl-2 and caspase 8 [11,18-20]. However, the HIV-1 PR-mediated cleavage of the anti-apoptotic protein Bcl-2, affecting the balance of anti- and pro-apoptotic proteins, was not confirmed by other laboratories [12,18,22]. In our experiments, Bcl-2 protein was not cleaved by HIV-1 PR. Caspase 8 was shown to be specifically processed by HIV-1 PR to a fragment termed Casp8p41 [18]. Full-length caspase 8 cleaves Bid to the C-terminal fragment tBid, the most important link between the extrinsic and intrinsic apoptotic pathways. The tBid translocates to the mitochondria to activate Bax/Bak channels and promote release of cytochrome c. However, as the Casp8p41 fragment generated by HIV-1 PR is catalytically inactive [19], the role of Casp8p41 in the activation of Bid is unlikely. Indeed, no Bid cleavage by HIV-1 PR was observed in our *in vivo* or *in vitro* experiments. Similarly, we failed to detect cleavage of pro-caspase 8 (Figure 5). Recent studies of the Casp8p41 fragment suggest that it could independently induce apoptosis *via* its interaction with mitochondria, resulting in rapid mitochondrial depolarization mediated by Bax/Bak, cytochrome c release and activation of caspase 9 and 3 [19,23,24]. These findings agree with our results showing that HIV-1 PR activates caspase 9 both *in vivo* and *in vitro* (Figure 5). The activation of caspase 9, but not caspase 8, was also observed in HIV-1 infected CD4+ lymphocytes [49].

Instead of proteolysis of pro- and/or anti-apoptotic factors, we observed a direct impact of HIV-1 PR on mitochondria. In the present study, we used several complementary methods to confirm that HIV-1 PR is associated with mitochondria: confocal microscopy showing co-localization of HIV-1 PR with a mitochondrial marker, detection of the presence of HIV-1 PR in mitochondria-enriched subcellular fractions, and immunogold labeling TEM. Although the mitochondrial localization of HIV-1 PR was rather unexpected, a previous report describes PR activity in the cytoplasm as well as in the membrane fraction of HIV-1-infected cells [9]. Active PR may also be released during uncoating of the viral core, in which it is present together with CA, NC, reverse transcriptase, RNase H, integrase, Vpr and Nef [50]. CA, NC and their fragments generated specifically by PR have been detected in an early stage of cell infection by EIAV and M-PMV [51,52].

Based on our results, we suggest that HIV-1 PR affects the integrity of mitochondrial membrane(s), which subsequently leads the cell to apoptosis. First, we visualized HIV-1 PR in the mitochondria both by confocal microscopy and TEM. Immunogold TEM and experiments based on controlled proteolysis of purified mitochondria by proteinase K showed that a portion of HIV-1 PR is localized below the mitochondrial surface, likely at the inner membrane. Interestingly, this mitochondrial localization was independent of HIV-1 PR activity. Second, we demonstrated that HIV-1 PR could efficiently process Tom22, VDAC and ANT *in vitro*, which could lead to a release of AIF and Hsp60 proteins *in vivo*. We therefore assume that active HIV-1 PR can cleave mitochondrial proteins, which would lead to loss of mitochondrial integrity. Third, the expression of HIV-1 PR caused loss of *in vivo* mitochondrial potential, which is one distinctive feature of the onset of programmed cell death. Generally, membrane potential changes are caused by opening of the mitochondrial permeability transition pore (MPTP). Fourth, we observed expression of HIV-1 PR-activated caspase 9, which is usually recruited and allosterically activated by the apoptosome formed by released cytochrome c and APAF-1. Once initiated, caspase 9 sets off the caspase cascade by processing pro-caspase 3. Activated caspase 3 then cleaves several cellular targets, including PARP and inhibitor of caspase-activated DNase (ICAD) allowing DNA fragmentation by CAD (Figures 4 and 5).

We show here that HIV-1 PR interacts with BCA3 in mitochondria and that coexpression of these proteins is connected with enhanced transcriptional activity of p53 on the *bax* promoter and enhanced apoptosis induced by HIV-1 PR. This finding is in very good agreement with the data published by Genini et al. [49]. They found that HIV-1 infection of CD4^+^ T cells led to p53 phosphorylation which is followed by increased level of p21, Bax and p53 proteins. Moreover, Genini et al. also demonstrated release of mitochondrial proteins cytochrome c and AIF as well as detectable alteration of mitochondrial membrane potential. Mitochondrial localization of BCA3 was described in association with two proteins, p73 and AIF [31,32]. The association of p73 and BCA3 in mitochondria leads to cell death [31]. Sastri et al. showed that BCA3 protein is up-regulated in response to oxidation stress and that it interacts with mitochondrially localized AIF [32]. It remains unclear whether the interaction of BCA3 with the HIV-1 PR occurs directly or whether it is mediated by some other protein. If the latter is the case, it is plausible that AIF fulfills the role of the interaction mediator. Our observation suggests that BCA3 could function as molecular regulator in response to cellular stress imposed by HIV infection.

## Methods

### Plasmid construction

All DNA manipulations were carried out using standard subcloning techniques, and plasmids were propagated in *E. coli* DH5α. All newly created constructs were verified by DNA sequencing. The full-length and spliced forms of the genes encoding human BCA3 and BAX were obtained by reverse transcription of total RNA from HeLa cells and cloned into pCMV-HA/c-myc (Clontech) or pET22b, resulting in *HA-BCA3, c-myc-BCA3* or *BAX* vectors, respectively. PCR fragments encoding M-PMV or HIV-1 protease (*PR),* their inactive forms *(D26N, D25N)* or HIV-1 protease precursor (*PRO*) were subcloned into plasmids for mammalian (pCMV-HA/c-myc) or bacterial (pET22b) expression, resulting in vectors *c-myc-M-PMV PR17 (D26N)*, *c-myc-M-PMV PR12 (D26N)*, *c-myc-HIV-1 PR*, *HA-HIV-1 PR*, *c-myc-HIV-1 PR (D25N)*, *HA-HIV-1 PR (D25N)c-myc-HIV-1 PRO*, and *HIV-1 PR pET22b*. Point mutations and deletions within *BCA3*, *HIV-1 PR*, and *BAX* genes were introduced by two-step PCR mutagenesis using primers carrying the desired mutations and suitable restriction sites. The PCR fragments were digested with appropriate restriction enzymes and ligated into HA/c-myc-pCMV or pET22b.

The *pSAX2* vector, which encodes HIV-1 proviral DNA with deletion of the *env* gene, was kindly provided by Dr. J. Luban. The *pGL3-bax-luc* luciferase reporter vector was kindly provided by Prof. L. Tuosto. The *pDsRed2-Mito* mammalian expression vector, which encodes a fusion of *Discosoma sp*. red fluorescent protein (DsRed2) and the mitochondrial targeting sequence from subunit VIII of human cytochrome c oxidase, was purchased from Clontech. Further details of the cloning strategy and full sequences of all PCR primers can be obtained from the authors upon request.

### Cell lines and protein expression

HEK-293 and HeLa cells were grown in Dulbecco’s modified Eagle medium (DMEM, Sigma) supplemented with 10% fetal bovine serum (Sigma) and 1% L-glutamine (PAA) at 37°C under 5% CO_2_. Typically, cells were plated at a density of 2–5 × 10^5^ cells/ml one day before transfection. The following day, cells were transfected with the appropriate plasmid using X-tremeGENE HP DNA Transfection Reagent (Roche) according to the manufacturer’s instructions. The cells were grown for 6–48 h post-transfection and washed with phosphate-buffered saline (PBS). Further processing depended on the type of experiment. A stable HEK-293 cell line expressing HA-BCA3 was prepared as described in [33].

### Expression and purification of HIV-1 PR

HIV-1 PR was purified as previously described [53]. Briefly, the vector *HIV-1 PR pET22b* was introduced in *E.coli* BL21 (DE3) RIL cells (Invitrogen) and HIV-1 PR was produced in the form of inclusion bodies. The inclusion bodies were isolated and resuspended in 60% acetic acid. The suspension was diluted with water and dialyzed against a chromatographic buffer (50 mM MES, 10% glycerol, 1 mM EDTA, 0.05% ME, pH 5.8). The refolded protein was then purified by ion-exchange chromatography (Mono S 5/50 GL, GE Healthcare). The appropriate fractions were aliquoted and frozen at −20°C.

### Western blotting

Proteins were resolved by reducing SDS-PAGE and blotted onto a nitrocellulose membrane. To prevent nonspecific interactions, the membrane was blocked with Blocking Solution (Thermo Scientific) and then incubated with primary antibody for 2 h to overnight. After washing with PBS (3×), the membrane was incubated with HRP-conjugated secondary antibody for 1–2 h at 4°C. The antigen-antibody complexes were detected by West Femto Chemiluminescent Substrate (Thermo Scientific) using a LAS-2000 imager. The following antibodies were purchased from Sigma-Aldrich: monoclonal anti-mitochondrial protein (MTC02) and rabbit anti- COX IV antibodies and anti-VDAC1/Porin from Abcam; monoclonal anti-HA peroxidase conjugate clone HA-7, EZview Red Anti-HA Affinity Gel, anti-alpha-tubulin (B512), anti-C11orf17(BCA3), anti-HA-TRITC, anti-HA clone HA-7 FITC, anti-c-myc FITC, and anti-p53 (PAb1801) antibodies and rabbit polyclonal anti-HSPD1, anti-AIF, anti-Tomm22, anti-c-myc, anti-VDAC/Porin, anti-CANX, and anti-BCA3 antibodies. The monoclonal anti-c-myc HRP, anti-c-myc (9E10), anti-Bax (2D2), and anti-HA (F-7) antibodies and goat anti-caspase 8p18(C-20) and anti-BCA3 (N-12) antibodies were purchased from Santa Cruz. The following antibodies were purchased from Cell Signaling; rabbit anti-Bcl2, anti-PARP, anti-Bid, anti-caspase 9, anti-Bax, and anti–acetyl-p53 (Lys382) antibodies and rabbit monoclonal anti-Bak (D2D3), anti-Bax (D2E11), anti-COXIV (3E11), and anti-cleaved caspase 8 (Asp391) antibodies and monoclonal anti-caspase-8 (1C12) antibody. Monoclonal anti-cytochrome C and anti-PKA(C) antibodies were purchased from BD Pharmingen; and rabbit anti-ANT antibody from LSBio. All secondary antibodies (anti-rabbit IgG, anti-mouse IgG) were HRP- (Sigma) or gold- (British Biocell) conjugated.

### Co-immunoprecipitation

Co-immunoprecipitation was carried out as previously described [54]. Briefly, HEK-293 or HeLa cells were grown on 60 mm plates and co-transfected with *HA-BCA3*, *c-myc-M-PMV PR17(D26N)*, *c-myc M-PMV PR12(D26N)* or *c-myc-HIV-1 PR(D25N)*. After 48 h, the cells were washed 1× with PBS and lysed in 300 μl CO-IP buffer B (20 mM Tris–HCl, pH 7.5, 150 mM NaCl, 1 mM EDTA, 1 mM DTT, 0.1 mM MgCl_2_, 0.5% NP-40) containing Halt Protease Inhibitor Mix (Thermo Scientific) for 30 min on ice. A 1/5 of the total cell lysate sample was used for Western blot analysis. The rest of the cell lysate was cleared by centrifugation and diluted with 1 200 μl CO-IP A buffer (20 mM Tris–HCl, pH 7.5, 150 mM NaCl, 1 mM EDTA, 1 mM DTT, 0.1 mM MgCl_2_). Primary antibody [monoclonal anti-c-myc (Santa-Cruz), rabbit anti-c-myc or EZview Red Anti-HA Affinity Gel (Sigma)] was added to the cell extract and incubated overnight at 4°C. Optionally, 20 μl Protein-A/G-Sepharose beads were added, and after 2 h incubation at 4°C, the immunoprecipitates were collected by centrifugation. The pellets were washed 3× with CO-IP buffer and then 1× with PBS. Proteins were resolved by SDS-PAGE and analyzed by Western blotting.

### Immunoprecipitation from mitochondrial and cytosolic fractions

HEK-293 cells (4×10^5^cells/ml) were seeded on 2×100 mm plates and co-transfected with *HA-BCA3pCMV* and *c-myc-HIV-1 PR(D25N)*. After 48 h, the cells were washed 1× with PBS and 1/20 of the cells was resuspended in PLB and used as total cell lysate sample for Western blot analysis. The rest of the cells was aliquoted: 1/4 was resuspended in 300 μl CO-IP buffer B, and immunoprecipitation was carried out as described above; 3/4 were resuspended in 0.5 ml homogenization medium HB (250 mM sucrose, 1 mM EDTA, 1 mM EGTA, 20 mM HEPES-NaOH, 10 mM KCl, 1.5 mM MgCl_2_, pH 7.4) supplemented with Halt Protease Inhibitor Mix (Thermo Scientific) and homogenized by 50 strokes in a Dounce homogenizer. The cell lysate was centrifuged for 10 min at 1000 × g. The pellet was resuspended in 0.5 ml HB, re-homogenized by 30 strokes in a Dounce homogenizer, and centrifuged for 10 min at 1 000 × g. Both supernatants were combined and centrifuged at 17 000 × g for 15 min. NP-40 and NaCl were added to the supernatant (cytosolic fraction) to final concentrations of 0.1% and 150 mM, respectively. The mitochondrial pellet was washed 1× with 400 μl HB and resuspended in 1 ml of CO-IP buffer A supplemented with 0.1% NP-40. 100 μl aliquots of both cytosolic and mitochondrial fractions were mixed with PLB and used as total cytosolic and mitochondrial lysate samples for Western blot analysis. 900 μl aliquots of both samples were used for immunoprecipitation using anti-c-myc and anti-HA antibodies, as described above.

### Subcellular fractionation and mitochondria isolation

To obtain a crude mitochondrial fraction, cells (1 × 10^8^ cells/ml) were washed with PBS, pelleted by centrifugation at 300 × g for 5 min and frozen at −70°C. The cell pellet was resuspended in 1 ml homogenization medium HB supplemented with Halt Protease Inhibitor Mix (Thermo Scientific) and homogenized by 50 strokes in a Dounce homogenizer. The cell lysate was centrifuged for 10 min at 1 000 × g. The pellet was resuspended in 1 ml HB, re-homogenized by 30 strokes in a Dounce homogenizer, and centrifuged for 10 min at 1 000 × g. Both supernatants were combined, and a crude mitochondrial fraction was obtained by centrifugation at 17 000 × g for 15 min. The pellet containing crude mitochondria was washed with HB (1×), resuspended in the same buffer and used for subsequent experiments. The remaining supernatant was subjected to ultracentrifugation at 100 000 × g for 1 h at 4°C, and the microsome-containing pellet was analyzed by Western blotting [55]. Mitochondria were further purified by centrifugation in a discontinuous gradient of Optiprep according to the manufacturer’s instructions (http://www.axis-shield-density-gradient-media.com). Briefly, the crude mitochondrial fraction was resuspended in 1.4 ml HB and 3.6 ml solution D [5 volumes of 50% iodixanol and 1 volume of solution C (0.25 M sucrose, 6 mM EDTA, 120 mM HEPES-NaOH, pH 7.4)]. This suspension was overlaid with 3.6 ml of each of two gradient solutions (with densities of 1.175 g/ml and 1.079 g/ml) and centrifuged at 50 000 × g for 3 h in a Beckman SW41 rotor. The gradient was collected in 1 ml fractions and analyzed by Western blotting. To recover purified mitochondria, the appropriate gradient fraction(s) was diluted by HB (1:3) and centrifuged at 17 000 × g for 15 min. Pelleted mitochondria were washed with HB and resuspended in HB solution. In experiments in which nuclear and mitochondrial fractions were isolated, the NE-PER Nuclear Extraction Kit and Mitochondrial Isolation Kit (Thermo Scientific), respectively, were used.

### HIV-1 PR treatment of cell lysates

HEK-293 or HeLa cells, optionally transfected with *HIV-1 Gag*, *c-myc-HIV-1 PR* and *HA-BCA3*, were washed with PBS, pelleted by centrifugation at 300 × g for 5 min and frozen at −70°C. The cell pellet was resuspended in 500 μl solution B (60 mM MES, pH 6.5, 120 mM NaCl, 0.1% NP-40) and homogenized by 50 strokes in a Dounce homogenizer. The cell lysate was centrifuged for 10 min at 1 000 × g. The pellet was resuspended in 500 μl 60 mM MES, pH 6.5, containing 120 mM NaCl, homogenized by 30 strokes in a Dounce homogenizer and centrifuged for 10 min at 1 000 × g. Both supernatants were combined and incubated with recombinant HIV-1 PR (1.25 μM final concentration), optionally in the presence of ritonavir (Sigma) (4 μM final concentration) or calpain inhibitor I (Sigma) (33–100 μM final concentration) at 37°C. The reaction was stopped by addition of 2× protein loading buffer (PLB). Samples were separated on 12 or 15% SDS-PAGE and analyzed by Western blotting.

### HIV-1 PR treatment of purified mitochondria

Freshly isolated mitochondria were diluted in SB buffer (200 mM sucrose, 10 mM HEPES-NaOH, pH 7.4, 2 mM NaCl, 5 mM KH_2_PO_4_, 2 mM MgCl_2_) to a final protein concentration of 1 mg/ml. Various amounts of recombinant HIV-1 PR (4 μM storage concentration) in the presence or absence of ritonavir (4 μM final concentration) were added, and incubation proceeded overnight at room temperature. The reaction was stopped by addition of 2× PLB. Proteins were separated by 12-15% SDS-PAGE and analyzed by Western blotting. In the experiments studying the release of the mitochondrial protein, the mitochondria were after incubation with HIV-1 PR centrifuged for 16 000 × g for 10 min at 4°C. The selected proteins from the supernatant were analyzed by Western blot.

### In vitro preparation and cleavage of Bax protein

The p21 and p18 forms of Bax protein were prepared by TNT Quick Coupled Transcription/Translation System (Promega) according to the manufacturer’s instructions. Briefly, 40 μl TNT Quick Master Mix was mixed with 1 μg plasmid DNA together with 2 μl ^35^S methionine (10 mCi/ml) in nuclease-free water to a final volume of 50 μl. The mixture was incubated for 3 h at 30°C. Buffer containing 60 mM MES, 120 mM NaCl, pH 5.5, (50 μl) was added to each sample, followed by treatment with HIV-1 PR (1.25 μM final concentration) in the presence or absence of ritonavir (8 μM final concentration) for various time at room temperature. The reaction was stopped by addition of 2× PLB. HIV-1 PR activity was monitored by cleavage of purified HIV-1 CANC protein. Samples were resolved by 15% SDS-PAGE and visualized by autoradiography.

### Proteinase K digestion of isolated mitochondria

The basic experimental procedure was adapted from previously described methods [56,57]. Freshly isolated mitochondria from 0.5 × 10^8^ HEK-293 cells co-transfected with *c-myc-HIV-1 PR(D25N)* and *HA-BCA3* were resuspended in 350 μl homogenization buffer without protease inhibitors, and protein concentration was assessed by Bradford assay. Fresh mitochondrial samples (300 μg/100 μl) were incubated (i) alone, (ii) in the presence of proteinase K (Sigma) (0.14, 1.4 or 14 μM final concentration) or (iii) in the presence of Triton X-100 (1% final concentration) and proteinase K (0.14, 1.4 or 14 μM final concentration) for 10 or 60 min on ice. The reaction was stopped by the addition of 6× PLB, and samples were analyzed by Western blotting.

### Luciferase assay

HEK-293 cells were grown in a 24-well plate and were transfected with the appropriate vectors. Cells were lysed in Glo™ Lysis Buffer (Promega) 48 h post-transfection, and luciferase activity was determined by One-Glo™ Luciferase Assay System (Promega) using a fluorescence reader. Relative luciferase activity was corrected for differences in transfection efficiency by co-transfection with a *GFPpCMV* construct followed by fluorescence measurement. Each transfection was performed in triplicate; at least three independent experiments were performed.

### Flow cytometry analysis of dead and apoptotic cells

Determination of dead cells was adapted from the procedure described by Vermes *et al.*[58]. HeLa cells growing in six-well plates were transfected with *c-myc-HIV-1 PR* at various times, and the cells were harvested at 12, 18, 24 and 48 h post-transfection by standard trypsinization [59]. The cells were gently washed twice in PBS (300 × g, 5 min), resuspended in annexin-binding buffer and labeled with FITC Alexa Fluor® 488 annexin V and propidium iodide (PI) according to the manufacturer’s protocol for Dead Cell Apoptosis Kit (Invitrogen). Analyses were performed by flow cytometry using BD LSR Fortessa with BD FACSDiva software. Ten thousand events were collected for each sample.

### Flow cytometry analysis of mitochondrial membrane potential (ΔΨm)

The protocol was adapted from previous reports [60,61]. HEK-293 cells were seeded in 12-well plates overnight and transfected with 0.5, 1 or 2 μg/ml *c-myc-HIV-1PR*. Changes in ΔΨm were determined by JC-1 fluorochrome (5,5′,6,6′-tetrachloro-1,1′,3,3′-tetraethyl benzimidazolylcarbocyanine iodide) stored in DMSO (Sigma) 24 h post-transfection. The cells were incubated in DMEM containing 12.5 μg/ml JC-1 at 37°C under 5% CO_2_ for 45 min. Cells incubated with 400 μM CCCP (carbonyl cyanide 3-chlorophenylhydrazone) stored in DMSO (Sigma) for 2 hours were used as a positive control. After incubation, the samples were washed twice with cold PBS, and cell pellets were resuspended in 500 μl PBS and immediately analyzed by flow cytometry using BD LSR Fortessa. Healthy cells containing red JC-1 aggregates were detected in the FL 2 channel, while green JC-1 monomers in apoptotic cells were monitored in the FL1 channel (FITC). Ten thousand events were collected for each sample.

### Fluorescence microscopy

HEK-293 or HeLa cells at a density of 1 × 10^5^ cells/ml were plated on a glass coverslip placed in a 6-well plate and incubated in DMEM medium supplemented with 10% FBS (Sigma) and 1% L-glutamine (PAA) at 37°C and 5% CO_2_. The following day, the cells were transfected with appropriate plasmid DNA using FuGENE HD Transfection Reagent (Roche) according to the manufacturer’s protocol. 48 h post transfection, the cells were washed with PBS, fixed with 4% formaldehyde and incubated for 30 min at room temperature. The cells were washed 2× with PBS and incubated 10 min in 0.4% Triton X-100, followed by 2 × 5 min incubation with 0.1% Triton X-100. The cells were then incubated with primary fluorescently labeled antibodies for 60 min at room temperature, washed 2× with PBS and mounted using mounting media (Vectashield, Vector Laboratories), containing or not DAPI (4′,6-diamidine-2-phenyl indole) at a final concentration of 1.5 μg/ml. The samples were then imaged using a laser scanning confocal microscope (Leica) or Olympus IX 81 microscope.

### Immunoelectron microscopy

#### Cell preparation

HEK-293 cells were fixed for 30 min at 4°C in 0.1 M PIPES, pH 7.5, containing 2% formaldehyde and 0.1% glutaraldehyde. After washing, the samples were gradually dehydrated at 4°C in 30%, 50%, 70%, 90% aqueous ethanol (10 min in each bath) and finally twice in anhydrous ethanol for 10 min. After dehydration, the samples were embedded in LR White. The cells were infiltrated at 4°C in a 1:2 (v/v) mixture of LR White and ethanol for 15 min, a 1:1 (v/v) mixture for 30 min and a 2:1 (v/v) mixture for 30 min, followed by overnight incubation in pure resin at 4°C. Polymerization was carried out at 18°C under UV light for 72 h.

#### Immunogold labeling

Ultrathin sections (90 nm) were mounted on parlodion-coated nickel grids. The grids were washed briefly in water and incubated in blocking buffer [1% BSA (w/v) in PBS, pH 7.4] containing 10% normal goat serum (v/v) for 25 min. The grids were then incubated in buffer A [0.5% BSA (w/v), 0.05% (v/v) TWEEN 20 in PBS, pH 7.4] containing monoclonal antibody against HA (Santa Cruz) or VDAC (Sigma), diluted 1:100, for 2 h at room temperature. The grids were washed three times in buffer A and then transferred to a droplet of goat anti-mouse IgG conjugated to 10 nm gold particles (British Biocell), diluted 1:25 in buffer A, and incubated 1 h at room temperature. After washing in buffer A, the grids were stained with uranyl acetate and examined with a JEOL JEM 1200EX electron microscope.

## Abbreviations

HIV-1: Human immunodeficiency virus 1; PR: Protease; PRO: Protease precursor; BCA3: Breast carcinoma-associated protein 3; AKIP-1: A-kinase interacting protein 1; M-PMV: Mason-Pfizer monkey virus; HA: Hemagglutinin tag; TEM: Transmission electron microscopy; PARP: Poly (ADP-ribose) polymerase; Tom22: Outer membrane translocase complex; VDAC: Voltage-dependent anion channel; ANT: Adenine nucleotide translocator; PKAc: cAMP-dependent protein kinase A; COX: Subunit of cytochrome c oxidase; OMM: Outer mitochondrial membrane; Bak: Bcl-2 homologous antagonist killer; Bax: Bcl-2-associated X protein; Bcl-2: B-cell leukemia/lymphoma 2; CA: Capsid protein; NC: Nucleocapsid protein; AIF: Apoptosis inducing factor; Hsp60: Heat shock protein; PI: Propidium iodide.

## Competing interests

The authors declare that they have no competing interest.

## Authors’ contributions

MR conceived the ideas, designed the experiments, performed the experiments and prepared the manuscript, IK performed confocal microscopy and tissue culture experiments, RH performed EM, AK performed experiments with isolated mitochondria and flow cytometry, KS and MD performed some experiments, MH designed flow cytometry experiment and evaluated the results, IP contributed in manuscript preparation, TR conceived the ideas and contributed in manuscript preparation. All authors read and approved the final manuscript.

## Supplementary Material

Additional file 1: Figure S1 Association of HIV-1 PR, its various forms and BCA3 with mitochondrial membranes. (a) Detailed analysis of the composition of mitochondrial fractions 1–12 from HeLa cells expressing HIV-1 PR(D25N) and BCA3. Fractions from Optiprep gradient ultracentrifugation of HeLa cell lysates were analyzed by Western blot using the antibodies indicated. (b) Schematic representation of HIV-1 PR constructs used in this work. ^25^DTG^27^ is the active site triplet; TFP is the transframe octapeptide that forms the native N-terminal flanking sequence together with p6. (c) Mitochondrial localization of HIV-1 PR variants. HEK-293 cells were transfected with indicated constructs expressing various forms of HIV-1 PR, optionally in the presence of ritonavir (final concentration of 10 μM). Mitochondrial fractions 1–12 from the Optiprep gradient were analyzed by Western blotting. (d) Detection of BCA3 and HIV-1 PR in ER fractions. The supernatant remaining after isolation of mitochondria from the cells expressing BCA3 and HIV-1 PR (D25N), described in Material and Methods, was subjected to ultracentrifugation at 100 000 × g for 1 h at 4°C, and the microsome-containing pellet, highly enriched in ER bur largely devoid of mitochondria [55] was analyzed by Western blotting using anti-calnexin antibody as a marker of ER. (e) Immunoprecipitation of HIV-1 PR(D25N) and HIV-1 PRO(D25N) from BCA3 stable transfected cell line (D1): D1 cells were transfected with *c-myc-HIV-1 PR (D25N)* and *c-myc-HIV-1 PRO (D25N)*. 48 h post transfection the cells were lysed and 1/10 of the lysate was analyzed using anti-c-myc (upper panel) and anti-HA (lower panel) antibodies. The rest of cell lysate was divided into two halves: one half was immunoprecipitated with anti-c-myc and the second with anti-HA antibodies. Precipitates were blotted and developed with anti-c-myc (upper panel) or anti-HA (lower panel) antibodies. Click here for file
