# Supplementary figures and images for: HIV-1 protease-induced apoptosis
Source: Retrovirology. 2014 May 20;11:37. doi: 10.1186/1742-4690-11-37 (PMC4229777; doi:10.1186/1742-4690-11-37)

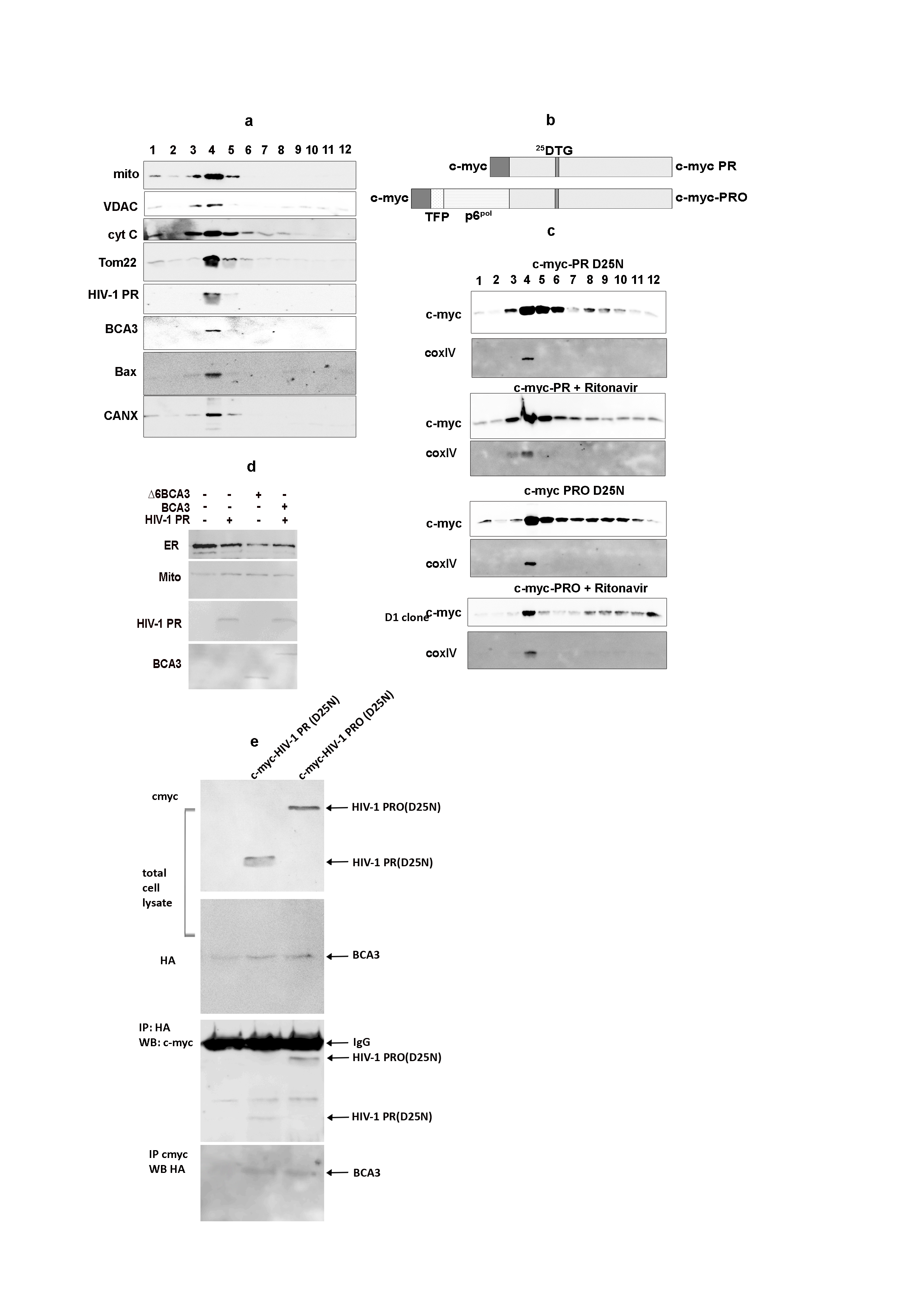

Supplement: Additional file 1: Figure S1 — Association of HIV-1 PR, its various forms and BCA3 with mitochondrial membranes. (a) Detailed analysis of the composition of mitochondrial fractions 1–12 from HeLa cells expressing HIV-1 PR(D25N) and BCA3. Fractions from Optiprep gradient ultracentrifugation of HeLa cell lysates were analyzed by Western blot using the antibodies indicated. (b) Schematic representation of HIV-1 PR constructs used in this work. 25DTG27 is the active site triplet; TFP is the transframe octapeptide that forms the native N-terminal flanking sequence together with p6. (c) Mitochondrial localization of HIV-1 PR variants. HEK-293 cells were transfected with indicated constructs expressing various forms of HIV-1 PR, optionally in the presence of ritonavir (final concentration of 10 μM). Mitochondrial fractions 1–12 from the Optiprep gradient were analyzed by Western blotting. (d) Detection of BCA3 and HIV-1 PR in ER fractions. The supernatant remaining after isolation of mitochondria from the cells expressing BCA3 and HIV-1 PR (D25N), described in Material and Methods, was subjected to ultracentrifugation at 100 000 × g for 1 h at 4°C, and the microsome-containing pellet, highly enriched in ER bur largely devoid of mitochondria [55] was analyzed by Western blotting using anti-calnexin antibody as a marker of ER. (e) Immunoprecipitation of HIV-1 PR(D25N) and HIV-1 PRO(D25N) from BCA3 stable transfected cell line (D1): D1 cells were transfected with c-myc-HIV-1 PR (D25N) and c-myc-HIV-1 PRO (D25N). 48 h post transfection the cells were lysed and 1/10 of the lysate was analyzed using anti-c-myc (upper panel) and anti-HA (lower panel) antibodies. The rest of cell lysate was divided into two halves: one half was immunoprecipitated with anti-c-myc and the second with anti-HA antibodies. Precipitates were blotted and developed with anti-c-myc (upper panel) or anti-HA (lower panel) antibodies. [file 1742-4690-11-37-S1.tiff]
